# The Assessment of Vision in Children with Severe Learning Difficulties: A Systematic Review

**DOI:** 10.22599/bioj.324

**Published:** 2024-03-27

**Authors:** Hareem Esmail, Gemma Arblaster, Laura Haslam

**Affiliations:** 1 Orthoptic Department, The Newcastle upon Tyne Hospitals NHS Foundation Trust, Newcastle, UK; 2 School of Allied Health Professions, Nursing and Midwifery, Faculty of Health, University of Sheffield, UK; 3 Orthoptic Department, Sheffield Teaching Hospitals NHS Foundation Trust, Sheffield, UK; 4 School of Allied Health Professions, Nursing and Midwifery, Faculty of Health, University of Sheffield, Sheffield, UK

**Keywords:** vision assessment, learning difficulties

## Abstract

**Background:**

Children with learning difficulties that require a vision assessment may not be able to perform standard clinical vision tests, for example, Forced Choice Preferential Looking (FCPL). There is a lack of standardisation on the procedure of vision assessment in this group of children. The aim of this literature review was to identify and evaluate methods of vision assessment when standard clinical vision tests are not possible in children with severe learning difficulties.

**Method:**

Three databases (CINAHL, PubMed, Web of Science) were searched from inception to Nov 2022 for methods of vision assessment in children with learning difficulties. Reference lists and grey literature were also searched. The McMaster University Critical review form for quantitative studies was used to assess the methodological quality of the primary studies identified.

**Results:**

Five-hundred and seventy one papers were identified from databases and 16 were identified from searching reference lists and grey literature. Of the 587, five studies were relevant and fulfilled all the inclusion and exclusion criteria. Three methods of vision assessment were identified: Visually Evoked Potentials (VEP), questionnaires, and the Bradford visual function box (BVFB).

**Discussion:**

The VEP method was validated and reliable, although it had a similar success rate to the standardised FCPL tests in children with learning difficulties. The BVFB was a standardised method for measurement of vision threshold in children that cannot successfully complete FCPL tests, however it has not been validated. Questionnaires are an efficient way to gather descriptive information on the child’s functional vision, however no guidance on the interpretation of the information is available. The BVFB and questionnaires require further development and validation. All three methods (VEP, questionnaires, and BVFB) can be useful as part of the assessment of vision in a child with severe learning difficulties where standard clinical tests are not possible, when used in a standardised manner.

## Background

Children with learning difficulties are at an increased risk of having ocular disorders such as strabismus, refractive error, cerebral visual impairment (CVI), optic atrophy, retinopathy of prematurity, and accommodative disorders (Salt and Sargent 2014). A cohort of 923 Danish children with developmental delay (borderline to severe) (aged 4–15 years) were reported by Nielsen et al. (2007a). 10.5% had visual impairment, but this increased to 22.4% in those with severe developmental delay (IQ < 50). The most common aetiologies of visual impairment were CVI, optic atrophy, and nystagmus. A follow-up paper of the same cohort found 44% also had clinically significant refractive error (Nielsen et al. 2007b). It is important for children with learning difficulties to undergo visual assessment(s) as visual impairment can have a significant negative impact on a child’s ability to learn and develop (Dale and Sonksen 2002). CVI can often go undiagnosed in childhood, which may be due to a lack of suitable assessments of CVI. Chokron et al. (2021) reported it may also be due to a lack of awareness of the condition and the focus of their care being on the child’s behavioural and learning disorders.

Children with learning difficulties may have their vision assessed in an orthoptic clinic or by an Orthoptist in a school setting. Due to limited intellect, ability and/or engagement (with tests), there may be difficulty performing visual acuity (VA) tests that children of the same age without learning difficulties can perform. Nielsen et al. (2007a) reported 2.5% of children with developmental delay and 5.8% of children with severe developmental delay were unable to perform any standard VA testing. This included letter and number optotypes, Cardiff Acuity Cards and Teller acuity cards depending on the child’s ability. Das et al. (2010) assessed VA using standardised vision testing methods in 240 children with physical and/or complex intellectual disabilities from six special needs schools in Glasgow. One hundred and eighty three had learning difficulties and 38 (21%) were unable to complete VA testing due to limited engagement.

Vision assessment in children with learning difficulties typically includes both visual function and functional vision. Visual function assessment quantitatively measures vision to determine threshold measurements e.g. VA, contrast sensitivity, colour vision, and visual fields. Functional vision assessment qualitatively evaluates the individual’s visual ability or how they use their vision. In patients where a threshold VA measurement is unsuccessful with Forced Choice Preferential Looking (FCPL) or other standardised clinical tests, vision is often assessed by non-standardised means. For example, visual responses and fixation may be assessed using a torch or a toy at various distances. These observations and descriptions can lack accuracy and repeatability, limiting clinical value to reliably assess and detect change in vision. A British and Irish Orthoptic Society survey (2018) of the Special Education Needs (SEN) Special Interest Group (SIG) members (n = 341) gathered information on SEN services. Thirty two responses were received from SEN SIG members. Only 12 reported having standardised methods for the functional assessment of vision in children with profound learning difficulties. However, no details of the standardised methods were provided in the survey report (British and Irish Orthoptic Society 2018).

Paediatric ophthalmologists have reported vision assessments in children with learning difficulties can help to gain information relevant to their management (Morale et al. 2012). Parents of children with developmental disabilities and visual impairment have been reported to use VA results to visualise and guide their selection of object and toy sizes when visually engaging their child (Lehman 2013). Morale et al. (2012) demonstrated that clinician and parent discussion about VA results achieved (Teller acuity cards) in children with learning difficulties (n = 309) increased parental knowledge and significantly reduced parental concerns about their child’s vision.

The aim of this literature review was to identify and appraise methods available to quantitatively measure VA and/or qualitatively assess functional vision in children with severe learning difficulties unable to perform standard VA testing, the most basic of which is the FCPL method.

## Methodology

A systematic search of the medical literature was performed using three literature databases PubMed (1966–20/11/2022), CINAHL (1981–20/11/2022) and Web of Science (1900–20/11/2022). Reference lists from the primary papers, books and relevant systematic reviews and grey literature were also searched to identify relevant literature. Search terms are shown in Table 1. Sources were included if they reported children or young people (0–25 years old) with moderate to severe learning difficulties or children or young people unable to perform a standard VA test (such as FCPL or Cardiff Acuity Cards) in any setting (health or education). Language was not restricted. Sources were excluded if they reported visual assessment in a specific learning difficulty such as dyslexia, assessment of adults only, assessment of children with normal intellectual development or mild learning difficulties only, or the visual assessment required subjective responses from the patient (such as pointing at an optotype or making a large head movement to indicate a stimulus had been seen).

**Table 1:** Search terms used in the systematic search of the literature.


POPULATION	EXPOSURE	OUTCOME

Child* “young person” “young patient*” “children and young people” CYP “paediatric patient*” “pediatric patient*” “special school*” student* “intellectual difficult*” “intellectual disabilit*” “learning difficult*” “learning disabilit*” “neurological impairment” “developmental delay” “developmentally delayed” “complex needs” “complex disabilit*” “special needs” “special education needs” “special educational needs” SEN “multiple needs” “cognitive impairment” “Cerebral Palsy” “Down syndrome” “brain injury” “preterm birth” Premature	“vision assess*” “visual assess*” “visual acuity test*” “vision test*” “visual function test*” “functional vision test*” “visual function assess*” “functional vision assess*” “visual ability”	vision “visual acuity” “visual function” “color vision” “colour vision” “visual field” “contrast sensitivity” “functional vision” “visual impairment” “vision impairment” VI “cerebral visual impairment” CVI

Population terms combined with OR Exposure terms combined with OR Outcome terms combined with OR Search used Population terms AND Exposure terms AND Outcome terms		


A data extraction template was designed based on guidelines from the Centre of Reviews and Dissemination (Akers et al. 2009). Data was extracted on the characteristics of the study, participants and methods of assessment. Methodological quality was assessed using the McMaster University Critical Review Form (Law et al. 1998).

## Results

**Figure 1:**
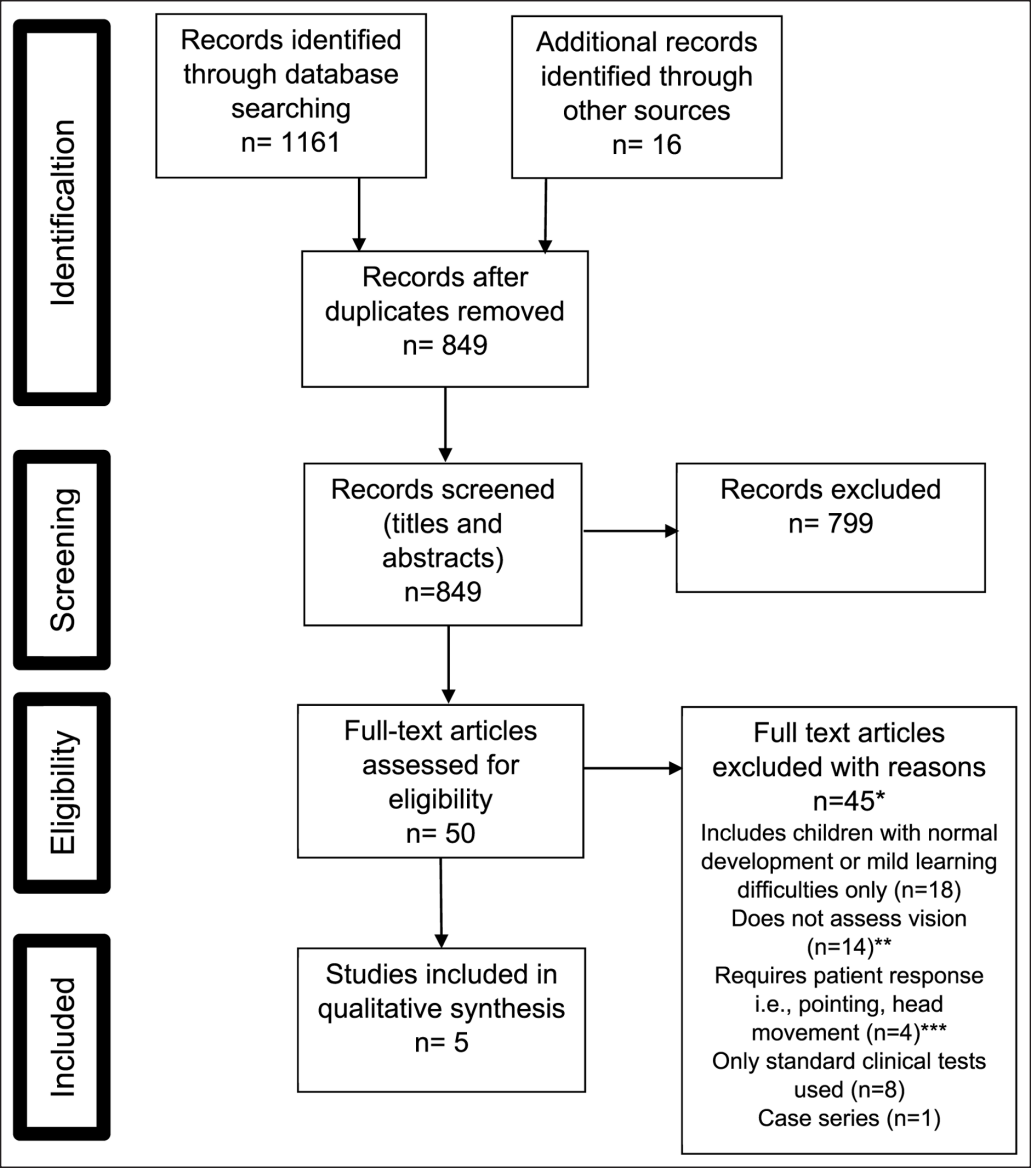
PRISMA flow diagram depicting the different phases of the systematic literature search. * One article read for full text assessment was in Dutch language. ** Excluded as did not report vision testing of patients, for example described the development of a vision assessment tool only. *** Excluded as children with intellectual ability to provide these responses should be able to perform FCPL tests.

The systematic search returned 1161 citations from databases. Sixteen additional citations were identified through searches of reference lists. Five studies met the inclusion criteria. Figure 1 shows the search and selection process.

### Study

#### Study purpose

Three methods of visual assessment were identified. Two studies compared Visually Evoked Potentials (VEP) with FCPL tests (Good 2001, Mackie et al. 1995). McCulloch et al. (2007) introduced the Visual skills inventory questionnaire, responses of which were compared to VEP and FCPL tests. Ferziger et al. (2011) also evaluated their Functional visual questionnaire. Pilling et al. (2016) reported the Bradford visual function box (BVFB).

#### Study designs

Four studies of the five included were cross-sectional studies which is the most appropriate study design to compare the outcomes of the proposed visual assessments to currently standardised VA tests. The remaining was a case study design which reported outcomes and inter-tester reliability of a proposed visual assessment (BVFB) in children with learning difficulties (Pilling et al. 2016).

Data was collected from a convenience sample in all 5 studies. The sample size ranged from 22 (Pilling et al. 2016) to 77 (Ferziger et al. 2011). No justification was given for the sample size in any study. Inclusion criteria was given for only two of the studies (Ferziger et al. 2011; Good 2001). A summary of the five studies analysed can be found including the objective, inclusion/exclusion criteria and details of the validity and reliability in Appendix 1.

#### Participants

Good (2001) only recruited participants with a diagnosis of cortical visual impairment. General diagnoses of participants included: cerebral palsy (Ferziger et al. 2011), central nervous system injury (Good 2001) and handicaps caused by ischemic insult, prematurity, congenital and infections (Mackie et al. 1995). Levels of learning difficulties ranged from normal to severe in two of the studies (Mackie et al. 1995, McCulloch et al. 2007) whereas the others had only recruited children with severe, profound, or complex learning difficulties. Four studies included children with a range of abilities, from children that could successfully complete standardised VA tests to children that could not comply with standardised VA testing due to severe learning difficulties (Ferziger et al. 2011; Good 2001; Mackie et al. 1995; McCulloch et al. 2007). Pilling et al. (2016) reported children with severe learning difficulties who were unable to perform FCPL. Results for children with the various levels of learning difficulties were presented collectively, therefore, it was not possible to extract data only from children with severe learning difficulties that could not comply with standardised VA tests.

#### Methods of assessment

The identified methods of visual assessment were quantitative (VEP and BVFB) and qualitative (questionnaires).

#### Quantitative assessment – VEP

McCulloch et al. (2007) and Mackie et al. (1995) used the pattern onset VEP technique and Good (2001) used the sweep VEP technique. Both techniques can be used to assess infants and children with poor fixation (Odom et al. 2016; Almoqbel et al. 2008).

**Figure 2:**
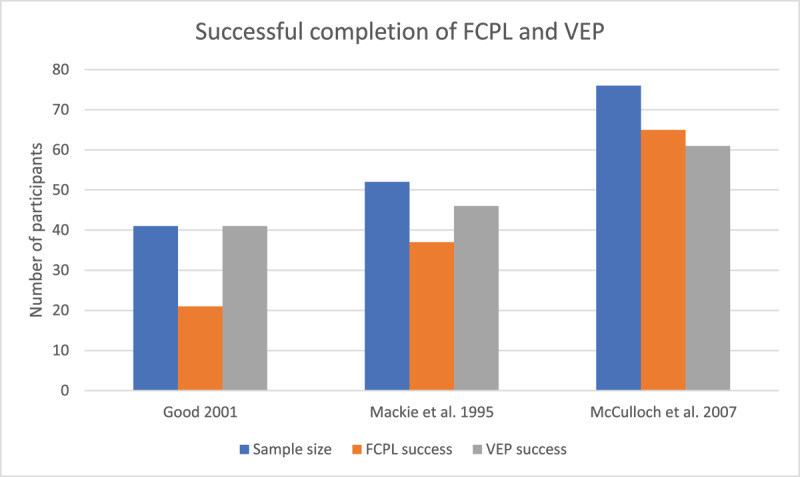
Comparison of the three studies reporting the number of participants successfully completing VEP and FCPL testing.

Good (2001) and Mackie et al. (1995) reported a good correlation between VEP and FCPL test (r^2^ = 0.662, P = 0.0003, and r^2^ = 0.34, p < 0.02 respectively). McCulloch et al. (2007) reported good agreement between FCPL and VEP results (tau = 0.47, p < 0.001). Good (2001) found higher VA with VEP compared to FCPL whereas Mackie et al. (1995) found lower VA with VEP compared to FCPL. McCulloch et al. (2007) found 80% of the children successfully completed VEP testing and 86% completed a FCPL test. They noted children with higher intellect, which was determined by the paediatric neurologist, were more likely to successfully complete the FCPL test, however intellectual ability did not determine success in VEP testing. Mackie et al. (1995) reported that 60% of the children with severe learning difficulties successfully completed the FCPL, compared to 100% that completed a VEP. No significant difference in success rates for completion of FCPL and VEP was found in the other groups (normal neurological development, mild moderate learning difficulties) studied by Mackie et al. (1995). Figure 2 displays the result from the three studies that compared VA with FCPL and VEP (Good 2001; Mackie et al. 1995; McCulloch et al. 2007). Good (2001) used Teller acuity cards for FCPL testing while Mackie et al. (1995) and McCulloch et al. (2007) used Keeler acuity cards. It is assumed that all studies used the testing procedure recommended by the manufacturers of each test. However, it is noted that Good (2001) reported the Teller acuity cards were held at 1m. It is therefore assumed that the VA reported was converted to account for this test distance.

#### Quantitative assessment – BVFB

The BVFB was developed as a visual function measurement tool for children with profound learning difficulties (Pilling et al. 2016). Vision threshold is determined from the smallest object the child can respond to from the BVFB (n = 11) and a score of their response (0 = uncertain of response to 3 = very certain of response). For each child (n = 22), two practitioners measured VA threshold and graded their responses. Good interrater reliability and a 100% success rate of using the BVFB to measure vision function was reported. Pilling et al. (2016) also presented a detailed list of questions for clinicians to use for the purpose of history taking and gathering parental or teacher’s observations of the child’s vision giving the clinician a broader picture of the child’s visual function. It was also implied that the BVFB can be used to assess visual fields to confrontation however, no visual field assessment results were presented.

#### Qualitative assessment – questionnaires

Two studies evaluated questionnaires as a complementary method to vision assessment in children with learning difficulties (Ferziger et al. 2011; McCulloch et al. 2007). The questionnaires aimed to provide additional information on visual behaviour and were not designed as standalone methods of assessing vision. A summary of the characteristics of the questionnaires is provided in Appendix 2.

The Visual Skills Inventory (McCulloch et al. 2007) was sent to the homes of 126 children for parents/carers to complete prior to their clinic appointment. Data was presented for children with varying levels of learning difficulties who had returned the inventory and attended the clinic appointment (n = 76). Forty-six of 76 (62%) had fully completed the questionnaire.

The Functional Vision Questionnaire was developed to assess daily visual performance in children with Cerebral Palsy (Ferziger et al. 2011). All children had severe motor and neurological impairment. Questionnaires were completed by a primary educator following a 2-week observation period with a later clinical assessment of vision. Clinical data (n = 77) and questionnaire data (n = 47) were presented.

Both studies reported the refinement of the respective questionnaires using exploratory factor analysis. The Visual Skills Inventory results were compared to VEP results and both questionnaires were compared to FCPL to assess validity. The Functional Vision Questionnaire was tested for intertester and test-retest reliability.

#### Ordinal vision scales

**Figure 3:**
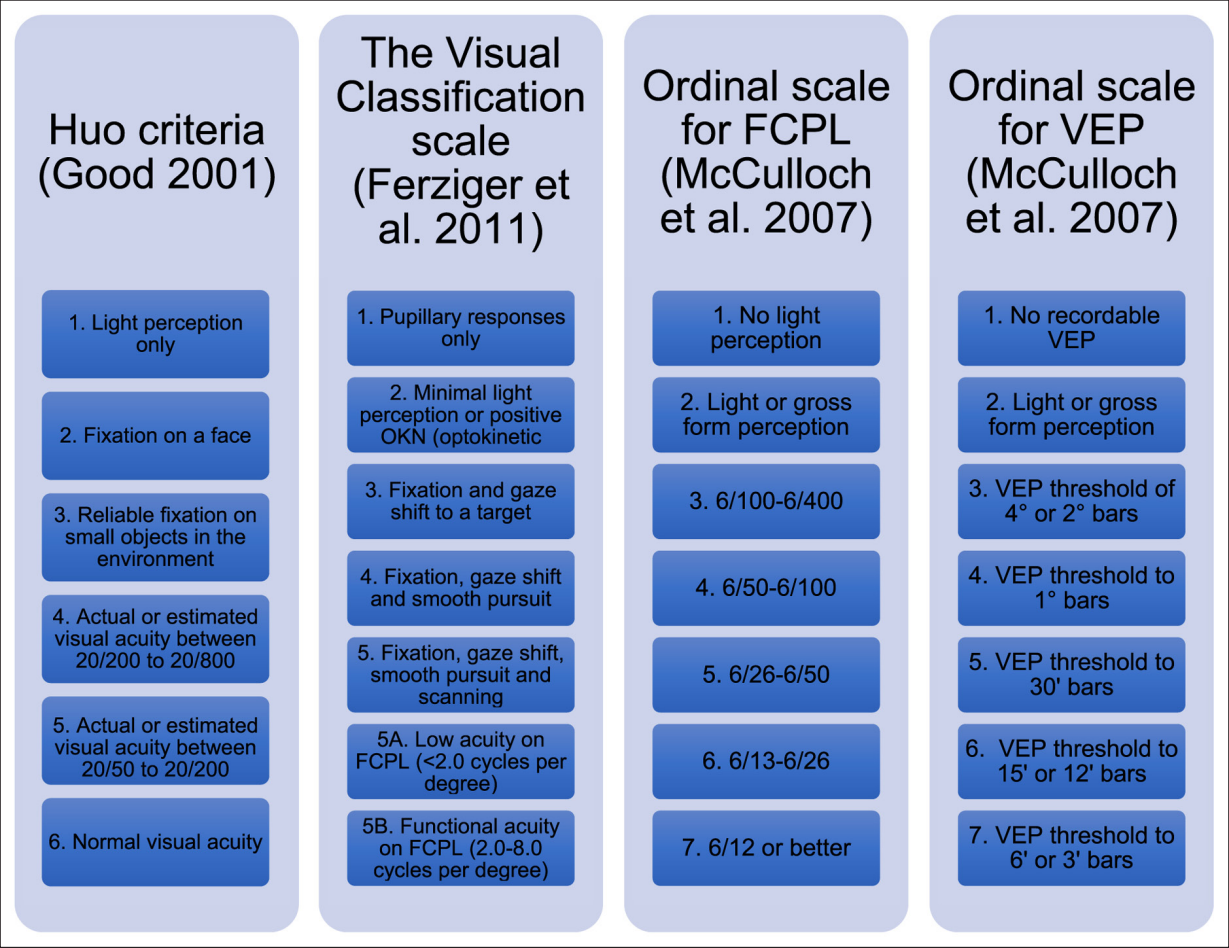
Ordinal scales used to classify vision VEP = Visually Evoked Potentials FCPL = Forced Choice Preferential Looking.

Three of the five studies used an ordinal scale to classify level of vision as part of their analysis. These scales are shown in Figure 3.

## Discussion

This literature review aimed to identify methods for the assessment of vision in children with moderate to severe learning difficulties, where a standard clinical VA test was not possible. A systematic search of the literature has identified three methods of vision assessment, two of which are alternative methods to a standard VA test (such as FCPL), VEP (Good 2001; Mackie et al. 1995), BFVB (Pilling et al. 2016) and one, using questionnaires, is complementary to a VA test (Ferziger et al. 2011; McCulloch et al. 2007). In a child with learning difficulties assessment of both visual function and functional vision are important. The VEP and BVFB methods quantitatively measure visual function (visual acuity threshold i.e. the smallest target that can be distinguished). Questionnaires qualitatively assess functional vision (i.e. performance at visual tasks).

### VEPs

Electrodiagnostic techniques to measure VEPs have been available for a number of years and standards are available for testing VEPs (Odom et al. 2016). Despite this, VEPs are not used routinely to measure VA threshold in children with moderate to severe learning difficulties, possibly due to cost, accessibility, the testing modifications required for this population (Odom et al. 2016), or due to the difficulty in consistently interpreting VEP measured acuity compared to clinical assessments of vision in all cases (Zheng et al. 2020). A VEP measurement of VA threshold does not require higher cognitive function, instead it measures visual cortex responses to a visual stimulus (Hamilton et al. 2021), which may explain the higher rate of successful completion of a VEP compared to a FCPL VA test (Mackie et al. 1995). VEPs do not assess functional vision, but they are an effective and accurate method of measuring VA threshold, with reliable, repeatable, and validated results (Good 2001; Mackie et al. 1995). The mixed results compared to FCPL, such as VEPs measuring higher VA than FCPL (Good 2001) and FCPL measuring higher VA than VEPs (Mackie et al. 1995) may have been due to a difference in testing order, patient tiredness, attention and/or engagement. These were not specified by Good (2001) or Mackie et al. (1995) and warrant further study. At present, the mixed results comparing VEP to FCPL results make it difficult for clinicians to interpret comparison between tests.

### BVFB

The BVFB vision assessment method (Pilling et al. 2016) has been used by others in a special school setting (Black et al. 2019), but has not undergone validity testing in comparison to another VA testing method (Appendix 1). Validity testing could include a comparison of the BVFB to a VEP or the BVFB to FCPL in a different cohort of children with learning difficulties, but who could perform a FCPL VA test. The BVFB is currently available for purchase; further details on the SeeAbility (2021) website.

### Questionnaires

The Visual Skills Inventory (McCulloch et al. 2007) and Functional Vision Questionnaire (Ferziger et al. 2011) assessed functional vision. Whilst the results are not quantified into a VA threshold, vision score or measurement, they are reported as a useful method to gather information about visual behaviour and functional vision. A survey carried out by BIOS (2018) showed that orthoptists within the UK and Ireland are using questionnaires for functional visual assessment and to aid the diagnosis of CVI. The questionnaires analysed in this review should be used in addition to a quantitative measurement of vision, rather than a standalone visual assessment tool. They aim to capture information describing functional vision observations in everyday life. Both questionnaires were reported as valid tools, following comparison of the results to VA measurements in children with learning difficulties that were able to perform FCPL (Ferziger et al. 2011; McCulloch et al. 2007) and VEPs (McCulloch et al. 2007). The Functional Vision Questionnaire had good interrater and test-retest reliability (Ferziger et al. 2011).

Advantages of the questionnaires included completion over longer observation periods in non-clinical settings in an attempt to gather information about visual abilities in naturalistic environments. However, this may have contributed to the limited completion rate of both questionnaires (Ferziger et al. 2011; McCulloch et al. 2007). While there are benefits of including a questionnaire as part of a clinical assessment, parents/teachers may be hesitant or may feel underqualified to assess or report vision. Reassurance may be required that questionnaires are only part of a vision assessment and are not diagnostic tools. Simultaneously, parents and teachers can be encouraged by explaining the value of their observations of the child throughout the day, compared to assessment in a clinical setting where the child may not be comfortable. Clear and detailed guidance on how to complete the questionnaires may be beneficial. The lack of questionnaire scoring and lack of guidance on clinical interpretation of the responses are problematic. Alternative vision questionnaires with scoring, such as the Visual Ability Score (VAS), have been developed (Katsumi et al. 1998). However, it has only been evaluated in children with ocular anomalies. At present the Visual Skills Inventory and Functional Vision Questionnaire results do not indicate normal or abnormal vision and no information is available on their reliability to measure or indicate change in functional vision.

Pilling et al. (2016) presented a checklist, in addition to the BVFB, containing questions similar to those in the Visual Skills Inventory and Functional Vision Questionnaire. The checklist was used to guide questioning of teachers or parents about their observations of the child’s visual ability to gather structured information. No specific data from the checklist was presented.

### Ordinal vision scales

Vision scales were used to classify vision from poor (lower value) to normal VA (higher value) in three of the studies (McCulloch et al. 2007; Ferziger et al. 2011; Good et al. 2001). Yet these scales were not evaluated as a measurement technique. One was created for the research (McCulloch et al. 2007). The Vision Classification Scale (Hoyt 2003) was used by Ferziger et al. (2011) and the Huo criteria (Huo et al. 1999) was used by Good (2001). No validity or reliability of these vision scales was reported, instead they were used to combine quantitative and qualitative assessments of vision into one description, which is similar to a low vision scale (World Health Organisation 2021).

### Recommendations

There is a lack of standardisation in clinical practice when visual assessments in children with moderate to severe learning difficulties are undertaken (BIOS 2018). It is anticipated that a standardised method of vision assessment would improve the clinical accuracy of vision assessments in children unable to undertake a clinical VA test and improve the interpretation and understanding of the results by the parent or carer (Lehman 2013). Based on the evidence from this literature review, a standardised procedure for vision assessment in children with learning difficulties unable to complete a FCPL VA test should include the BVFB (or similar may be devised within the department) standardised measurement of vision in addition to VEP testing where possible. The lack of validation of the BFVB should be considered; however, on balance, it is the best clinical testing method currently and widely available. Questionnaires should be additionally used to gather information from parents, teachers or carers, to add information about functional vision and visual abilities in daily life. A VEP should be considered as a baseline VA test. Due to high cost and low accessibility of the VEP, repeat testing could be considered if a significant change in vision is suspected. One of the questionnaires should be incorporated into the assessment, however there is no evidence to support the benefit of one over another. Clinicians should consider whether the Functional Vision Questionnaire, completed by the primary educator, may provide more information about functional vision due to the 5-point response scale, compared to the binary responses (yes/no) of the Visual Skills Inventory, completed by parents. Questionnaires should be considered as excellent methods of gathering information about functional vision as observed by individuals that spend the most time with the child whilst they are in a known environment. Questionnaire responses could also be shared with the parent, teacher, qualified teacher of the visually impaired (QTVI) (if applicable) and other health professionals.

### Limitations

This systematic search of the literature was limited by including only five sources in the review, however this highlights the limited literature available on this area of clinical practice. It is possible that more studies may have been included in the review if populations or samples of children with severe learning difficulties were more clearly described or defined in the literature. It is also worth noting the difficulties in measuring the severity of learning difficulty.

A number of studies were excluded as they reported methods requiring subjective responses (Browder and Levy 1974), it was unclear whether a child with severe learning difficulties would be able to complete the test (Browder and Levy 1974), only children with mild learning difficulties were recruited (Newcomb 2010), or a method of vision assessment was presented without any evidence of testing on any children (Atkinson et al. 2002). Whilst the development of tools to assess vision is potentially helpful for this cohort of patients, further data reporting results in patients would support their use in a clinical setting and add to the available evidence. Tsai et al. (2022) presented the Visual function battery for children with special needs (VFB-CSN); a battery of scored tests assessing visual function (VA, contrast sensitivity etc.) and functional vision (how the vision is used), however this was not included in the analysis due to lack of clarity of the characteristics of the participants included.

Whilst all the included studies reported the assessment of vision in children with moderate to severe learning difficulties, a limited range of aetiologies were included in the patient cohorts. Ferziger et al. (2011), Good (2001), and Mackie et al. (1995) included children with learning difficulties due to brain injury or insult. McCulloch et al. (2007) and Pilling et al. (2016) did not provide the diagnosis of their cohorts. Care must be taken assuming that results gained from these studies apply to all children with severe learning difficulties. Further work to investigate a larger cohort with a wider range of diagnoses would provide valuable information as to whether the methods reported are usable and comparable in all patients with severe learning difficulties.

## Conclusion

A review of the literature on vision testing in children with severe learning difficulties has highlighted the lack of available literature to inform and support clinical practice. An attempt at standardising quantitative and qualitative visual testing in children who are unable to perform a standard clinical VA test should be made by using the BVFB (or similar) and VEP where possible. These can be combined with using questionnaires to gather information about functional vision, such as the Functional Vision Questionnaire and the Visual Skills Inventory. Further development and validation of the BVFB and the questionnaires is required.

## Appendices

**Appendix 1:** Summary of the studies (n = 5).

*PAPER*	OBJECTIVE	INCLUSION/EXCLUSION CRITERIA	VALIDITY	RELIABILITY

***Ferziger et al.*** (2011)	To evaluate the use of a functional visual questionnaire completed by primary educator as part of a vision assessment in children with cerebral palsy	Inclusion criteria: Diagnosis of cerebral palsy Diagnosed as having severe to profound motor and intellectual disabilities using developmental tests. Exclusion criteria: not given	Data from the two constructs identified by EFA was compared to the VCS Task-orientated visual function and VCS r = 0.802; 95% CI 0.669–0.885 Basic visual skills and VCSr = 0.691; 95% CI 0.504–0.816	Good interrater reliability (n = 34) (ICC = 0.873) Excellent test-retest reliability (n = 14) after 8 months (ICC = 0.988)

***Good*** (2001)	To assess the use of sweep VEP as a quantitative method of vision assessment in children with cortical visual impairment	Inclusion criteria: Diagnosis of cortical visual impairment Exclusion criteria: not given	Linear regression analysis showed correlation between VEP and Teller acuity cards (r^2^ = 0.64 P = 0.0005) Linear regression analysis showed correlation between VEP and Huo criteria (vision scale) (r^2^ = 0.63 P = 0.00004)	Good test-retest reliability (n = 23), linear regression analysis r^2^ = 0.662 significance level P = 0.0003

***Mackie et al.*** (1995)	To compare visual acuity thresholds achieved with grating acuity cards to VEP in multiply handicapped children	Inclusion criteria: not given Exclusion criteria: not given	Linear regression analysis showed correlation between VEP and FCPL tests (r^2^ = 0.34, significance level p < 0.02)	Not tested

***McCulloch et al.*** (2007)	To compare visual skills assessed by parents/carers of children with learning difficulties using the Visual skills inventory with a clinical vision assessment including VA test using FCPL and VEP	Inclusion criteria: not given Exclusion criteria: not given	Responses from each questionnaire item was compared to the FCPL and VEP results and “responses to most of the questions were associated with the level of VA”	Not tested

***Pilling et al.*** (2016)	To assess the use of a novel vision assessment tool (BVFB) in children with severe learning difficulties	Inclusion criteria: not given Exclusion criteria: not given	Not tested	Good intertester agreement- weighted Cohen’s kappa = 0.768.

VEP = Visually evoked potentials, VA = visual acuity, FCPL = forced choice preferential looking, BVFB = Bradford Visual function box EFA = exploratory factor analysis, VCS = Visual Classification scale, CI = Confidence interval, ICC = Intraclass correlation coefficient, P values presented as they appear in the data.

**Appendix 2:** Characteristics of questionnaires.

PAPER	NAME	PURPOSE OF QUESTIONNAIRE	MEASUREMENT FACTORS AS DISCOVERED BY EXPLORATORY FACTOR ANALYSIS	COMPLETED BY	RESPONSES	SCORING AND INTERPRETATION

Ferziger et al. 2011	Functional Vision Questionnaire	Description of vision skills	Task orientated visual skills, Basic visual skills	Primary educator	Items rated 1(never), 2(occasionally 25%), 3(some of the time 50%), 4(most of the time 75%), 5(often >75%) or N/A	No final score or instructions for interpretation

McCulloch et al. 2007	Visual Skills Inventory	Description of vision skills	Visual responses to food and objects, Visual responses to social content, Light perception and/or photophobia	Parent/carer	Binary (yes/no) responses for all. Four sub-questions with ordinal scale responses to rate degree of performance.	No final score or instructions for interpretation
